# Prehospital emergency medicine for children receiving palliative home care in Germany—a cross-sectional, exploratory study of EMS providers

**DOI:** 10.3389/fped.2023.1104655

**Published:** 2023-02-14

**Authors:** Holger Hauch, Naual El Mohaui, Vera Vaillant, Michael Sander, Peter Kriwy, Marius Rohde, Johannes Wolff, Daniel Berthold, Emmanuel Schneck

**Affiliations:** ^1^ Palliative Care Team for Children, University Children’s Hospital, Giessen, Hesse, Germany; ^2^ University Children’s Hospital Giessen, Department of Pediatric Oncology, Giessen, Hesse, Germany; ^3^ Department of Anesthesiology, Operative Intensive Care Medicine and Pain Therapy, University Hospital, Giessen, Hesse, Germany; ^4^ Institute of Sociology, University of Technology, Chemnitz, Saxony, Germany; ^5^ Department of Oncology, Cleveland Clinic, Pediatric Oncology, Cleveland, OH, United States; ^6^ University Hospital Giessen, Palliative Care Team for Adults, Giessen, Hesse, Germany

**Keywords:** emergency medical service, palliative home care, pediatric emergencies, cardiopulmonary resuscitation, do-not-resuscitate order

## Abstract

**Background:**

The prevalence of children with life-limiting conditions is rising, and since the amendment of the social insurance code in Germany, palliative home care teams have treated an increasing number of children. These teams provide 24/7 readiness, yet some parents still call the general emergency medical service (EMS) for various reasons. EMS is exposed to complex medical problems in rare diseases. Questions arose about the experiences of EMS and whether they felt prepared for emergencies involving children treated by a palliative care team.

**Methods:**

This study used a mixed methods approach to focus on the interface between palliative care and EMS. First, open interviews were conducted, and a questionnaire was developed based on the results. The variables included demographic items and individual experiences with patients. Second, a case report of a child with respiratory insufficiency was presented to assess the spontaneous treatment intentions of EMS providers. Finally, the need, relevant topics, and duration of specific training in palliative care for EMS providers were evaluated.

**Results:**

In total, 1,005 EMS providers responded to the questionnaire. The average age was 34.5 years (±10.94SD), 74.6% were male. The average work experience was 11.8 years (±9.7), 21.4% were medical doctors. Experience with a call of a life-threatening emergency involving a child was reported by 61.5% and severe psychological distress during such a call was reported by 60.4%. The equivalent distress frequency for adult patient calls was 38.3%. (*p* < 0.001). After review of the case report, the EMS respondents suggested invasive treatment options and rapid transport to the hospital. Most (93.7%) respondents welcomed the consideration of special training in pediatric palliative care. This training should include basic information about palliative care, an analysis of cases involving palliatively treated children, an ethical perspective, practical recommendations, and available (24/7) local contact for further guidance and support.

**Conclusion:**

Emergencies in pediatric palliatively treated patients were more common than expected. EMS providers perceived the situations as stressful, and there is a need for specific training with practical aspects.

## Introduction

As medical care improves, and children with life limiting conditions live longer, the prevalence of those increases. This is probably best documented in the UK (1). In Germany, the overall number of emergency medical service (EMS) responses and hospice and palliative care services has grown (2, 3). These conditions may have contributed to an unexpected number of EMS calls for children receiving palliative home care (4). Pediatric emergencies are a special challenge for the EMS since they are relatively rare, only 2%–10% of all emergency calls (5–7). The challenges for the EMS in pediatric calls can be categorized into themes, such as the special value of children, clinical difficulties with pediatric patients, and identification with the patient’s family (8). In the United States, a nationwide study of emergency medical technicians (EMTs)/paramedics, department physicians, and nurses revealed that these pediatric calls cause stress and anxiety and may lead to pediatric safety events (9). Potential problematic in EMS responses in a palliative home care setting is that paramedics and physicians are not regularly faced with chronically ill children with a complex history and often rare diseases (10, 11). Interviews with emergency staff, reported that managing pediatric cases was stressful to them; and that they did not feel sufficiently prepared (12).

Data on the specific aspects of emergency medicine in children receiving pediatric palliative care are lacking. Ambulance officers in Australia demanded practical support from pediatric palliative care specialists (13). The frequency of EMS calls involving children with LLCs is reported as 0.2% of all outpatient emergency operations (14). Furthermore, in emergencies, there are ethical challenges and decisions must be made in a very short time (15). The choice [e.g., for invasive ventilation or cardiopulmonary resuscitation (CPR)] can have profound consequences, such as long-term dependence on a respirator and high-risk admission to an intensive care unit. Regarding the interface between palliative care and emergency medicine, it is important to empower families to be prepared for emergencies. The advance care planning with a “Do-Not-Resuscitate (DNR)” order is essential to prevent non-indicated CPR, especially in children with terminal cancer and a very short life expectancy.

Since the introduction of specialized home palliative care (SHPC) in Germany (16), an increasing number of children and adolescents with LLCs are being treated at home (10). Palliative conditions are diverse and affect children with cancer, heart diseases, rare syndromes, trauma sequelae, asphyxia, metabolic diseases, and a large number from other pediatric subspecialties (10, 13, 17).

The treatment of pain, dyspnea, gastrointestinal symptoms, and anxiety are essential to ensure the stability of families and their psychosocial situation. In addition, physicians and nurses on SHPC teams are prepared for home visits 24/7.

Since the introduction of palliative home care, many parents have called the EMS. The likelihood of those calls is significantly increased with prolonged SHPC treatment, absence of a DNR-order, and a language barrier (4).

The Franco-German EMS aims to bring the doctor to the patient (18). An historical term used to summarize emergency care is the “rescue chain,” (19) which consists of two major parts: (1) prehospital care and (2) hospital care. There are differences in the strategies of the United States and Germany: in Germany, providers prefer to focus on prehospital care, whereas in the United States, providers focuses on the second step in the emergency departments of the hospital (20). However, these differences do not influence the patient’s perspective in a palliative situation (21). In Italy, a prospective multicenter study revealed that the relatives of adult patients with advanced cancer, called the EMS in 17.1% of cases because of uncontrolled symptoms or family distress despite enrollment in palliative home care (22). Data for children are still being generated; in our previous retrospective study, the rate of EMS calls was 11.6% for the SHPC cohort.

There are differences in palliative home care between children and adults (23), but the data are lacking regarding emergency calls. As a result of the previous data analysis (4), this follow up study focusses on the EMS.

The aim of this study was to evaluate the individual experiences of the EMS in pediatric and adult emergency calls. We assessed the challenges the providers were confronted with during these emergency calls and whether additional training was needed. Furthermore, the elements of a future specific training program were explored.

## Methods

We followed a multi-step process to obtain a better understanding of the interface between EMS providers and patients with life-limiting conditions. First, all EMS responses involving pediatric patients with life-limiting conditions were analyzed, as previously described (4). Factors that played a role in calling the EMS and data on the conducted EMS therapy and outcomes were aggregated to provide the EMS providers with feedback. In a next step, we conducted interviews with the supervisors of all EMS organizations in Germany: the German Red Cross, “Malteser Hilfsdienst,” “Johanniter Unfallhilfe,” and EMS service “Eschenburg”. We asked questions in an open format to assess their interest in collaboration and their awareness of problematic issues that EMS providers face when caring for patients with LLCs. We asked the supervisors whether their staff members saw the need for specific training. Additional questions determined whether their employees had reported problems with patients, especially children with LLCs. Based on these interviews, a questionnaire was developed and was sent to all emergency medical physicians (EMPs) and EMTs/paramedics in the area of the reporting pediatric SHPC team with 1.1 million inhabitants in Hesse, Germany. The demographic variables were age, gender, years of experience, worktime status, workplace, and education level. The questions had both structured and unstructured response formats.

### Experiences of EMS providers

We asked the participants whether they could remember an EMS call that involved a patient with an LLC. The EMS provider were asked to provide a free description of the last recalled response (adult or child), what problems the patient suffered from, and the treatment that was performed. The outcomes of the patients [i.e., transport to a hospital, outpatient therapy, performance of CPR, return of spontaneous circulation (ROSC), survival, and further care] were documented.

The perception of stress and the emotional burden of these EMS calls were recorded. In addition, the participants were interviewed about their self-reported perception of the confidence in managing an emergency concerning a patient with a life-limiting condition. In addition, we inquired whether the EMS providers had direct contact with a SHPC team.

### Suggestions for treatment in a fictitious case report

The participants were asked to evaluate a case with a 15-year-old female adolescent with hypoxic ischemic encephalopathy due to a perinatal asphyxia. The study participants were instructed to answer spontaneously when assessing the next diagnostic and therapeutic steps.

### Theoretical skills in palliative care

The participants were asked whether they were familiar with the SHPC team in their region. To assess their basic knowledge in palliative care, the participants were asked about the basic duties of the SHPC teams, whether patients have a legal claim for palliative home care, and whether EMS professionals felt responsible to treat patients with LLCs emergencies. In addition, the basic duties of the SHPC teams were evaluated.

### Demands, needs, and attitudes of EMS providers

We inquired whether the participants desired special training in palliative care and what topics and duration for this training seemed appropriate. We aimed to determine whether the participants felt responsible for patients with LLCs in an emergency.

### Study organization and statistics

Study participants could respond in paper form or participate online *via* a QR code (Lime Survey® GmbH, Hamburg, Germany). The data of the study participants were anonymized and assessed. An individual code for each questionnaire (coding: age, individual letters of the name, and one digit of the mobile phone number) excluded the possibility of double participation.

The study was approved by the Ethics Committee of the Justus Liebig University of Giessen, Hesse, Germany (file number: 88/2016). The German Registry of Clinical Studies approved this study (DRKS-ID: 00013318), and it was forwarded to the World Health Organization Registry for Clinical Trials.

Descriptive statistics were analyzed using SPSS 25.0 (IBM Corp. NY, USA). In the case of group-specific differences, statistical tests were performed. The Wilcoxon test was used to compare ranks if the variables were not normally distributed. The Chi-squared test was performed to assess for differences in distribution. A *p*-value <0.05 (two-tailed) was considered statistically significant.

## Results

### Open interviews with EMS supervisors

The open interviews with the supervisors of the EMS staff (=15) about issues faced by EMS providers with patients with LLCs revealed four main findings: (a) need for specific medical skills in palliative care, (b) emotional aspects, (c) pediatrics as an evergreen topic, and (d) identification of the EMS-SHPC interface as an important issue. Specific training in palliative care was considered important, and staff members expressed their willingness to integrate palliative care into the yearly EMS training program, with a special consideration of pediatric aspects.

- Pediatrics is not a focus of the education of EMTs/paramedics. In the study period, we identified three levels (durations) of EMS training: the basic level or “Rettungssanitäter” (520 h), “Rettungsassistent” (2 years), and “Notfallsanitäter” (3 years). For simplicity, all non-physician EMS personnel are referred to as “EMTs” in the following results. The topic “palliative care,” especially for children, was not recalled in any of the education materials. Furthermore, the rarity of the wide range of pediatric diseases causing life limitations as well as the intentions and experiences of SHPC teams were reported as unknown. Staff members were asked to collect information on palliative-based knowledge.
- All supervisors recalled more than one employee needing help to cope with pediatric emergencies. In most cases, the EMTs were parents of children at a similar age to the patient(s). Identification with the families/children was reported to play a major role for developing emotional distress. Supervisors demanded information about the emotional aspects of these cases and implications their employees.
- Because of the underrepresented educational attention and the uncertainty related to the pediatric aspects of emergency medicine, pediatrics as a whole was reported as important.

All Supervisors reported that effective communication between the EMS and palliative care team is needed. GPs, other specialized physicians, and children’s hospitals should provide medical files for the EMS at the children’s homes. A 24/7 network to communicate with palliative care providers was noted to be essential.

The key aspects (a–d) were used to create a questionnaire. In a second meeting with the supervisors, the results were discussed; after informed consent, the documents were sent to all EMTs and EMPs in the study region.

### Demographics

In total, 1,005 of all 1,200 (83.8%) EMS employees provided responses to the questionnaire that could be evaluated. Of these, 789 were EMTs (emergency medical technician/paramedic) and 216 were EMPs (emergency medical physicians) (Table 1). Male employees were more common in both occupational groups (EMT 75.8%; EMP 70.4%). EMPs were significantly older and had more work experience than EMTs. The scope of the work was different between the two groups; while 74.1% of the EMT work was full-time, 50.2% of the EMP work was fee-based. Regarding the education level of the study participants, all skill levels were represented.

**Table 1:** Study participants.

	Paramedics^a^ (EMT)	Emergency physicians (EMP)	Diff.
Number (*n*, %)	789 (78.5%)	216 (21.5%)	
Gender (%)	75.8 ♂ /24.2 ♀ MV (2)	70.4 ♂ /29.6 ♀ MV (1)	*p* < 0.001 (WRS-test)
Age (mean ± SD) (years)	33.4 ± 10.4 MV (2)	42.6 ± 9.5 MV (1)	*p* < 0.001 (WRS-test)
Work experience (mean ± SD) (years)	11.1 ± 9.6 MV (3)	14.3 ± 9.6 MV(1)	Ns
Scope of work (*n*/%)	576 (74.1%)	54 (25.8%)	*χ*^2^ (3) = 439.696
Full-time:	176 (22.7%)	$\begin{array}{c} \begin{array}{c} 38\;(19.2\text{\%})\;\\ 105\;(50.2\text{\%})\;\\ 10\;(4.8\text{\%})\;\\ \mathrm{MV}\;(7)\; \end{array}\}\; \end{array}$	*p* < 0.001
Part-time:	3 (0.4%)		
Fee-based:	22 (2.8%)		
Others:	MV (12)		
Level of training:	EMT (520 h): 227 (29.3%)	Resident: 54 (25.8%)	
	Paramedic (2-years): 411 (53.0%)	Fellow: 104 (49.8%)	
	Paramedic (3-years): 137 (17.7%)	Senior physician: 44 (21.1%)	
	MV: (14)	Chief physician: 7 (3.3%) MV: (7)	

MV (*n*), numbers of missing values; SD, standard deviation; Diff., statistically significant difference; ns, not significant, WRS, Wilcoxon Rank Sum- Test.

^a^ with the term paramedics all levels of EMS training duration (520 h, 2 years and 3 years) are included.

### Experiences of EMS providers

**Figure 1:**
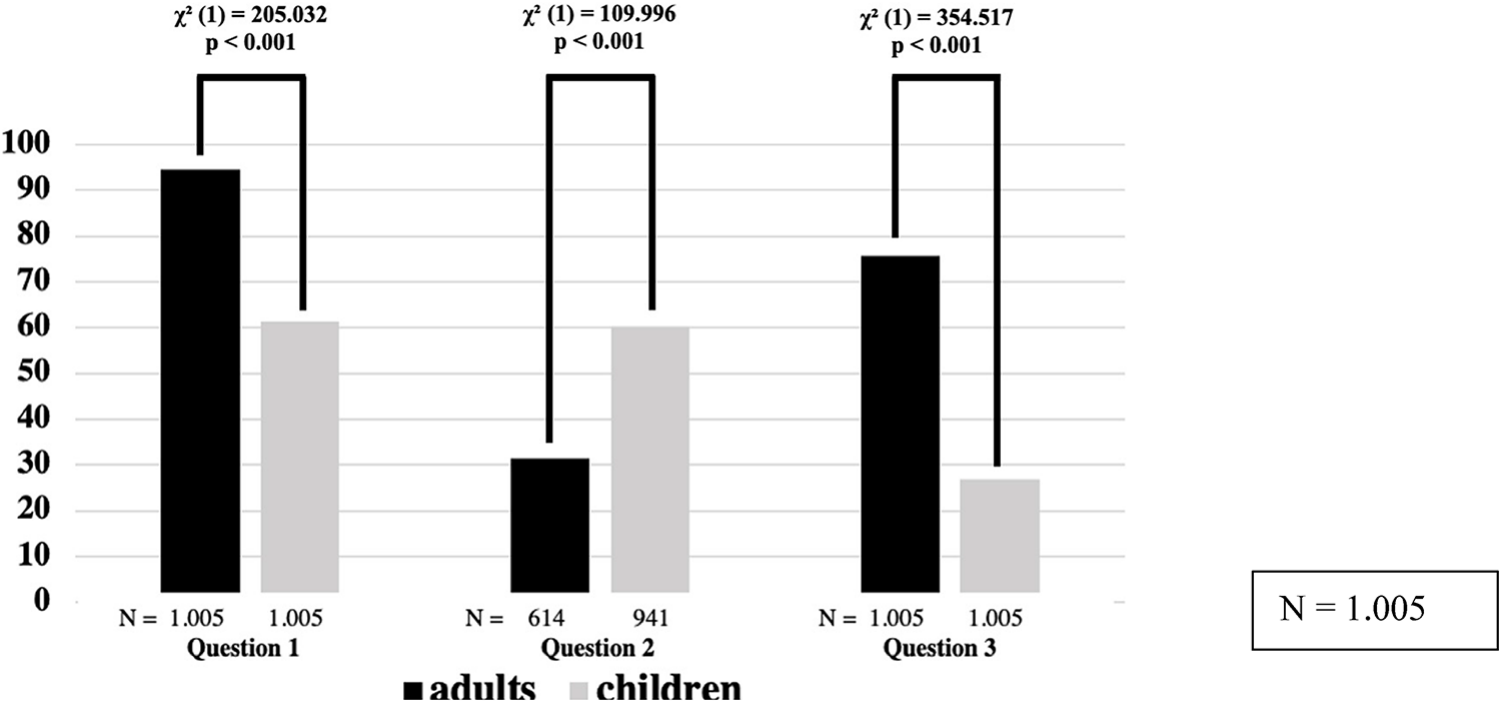
Experiences of participants. Q,question; LLC, life-limiting condition.

Almost all the study participants could recall at least one EMS call with a patient with an LLC (children: EMT 67.8%, EMP 47.8%; adults: EMT 96.1%, EMP 95.3%; *p* < 0.001) (Figure 1). The participants described significantly higher emotional burden and more uncertainties in dealing with seriously ill children compared with adults; 60.4% of all respondents felt overwhelmed in responses involving a child, whereas 31.4% felt overwhelmed in responses involving an adult (*p* < 0.001). There were no significant differences between the EMPs (63.4%) and the EMTs (57.3%) regarding feeling overwhelmed on calls involving children; however, there was a significant difference between the EMPs (39.2%) and the EMTs (29.0%) in regards to feeling overwhelmed on calls involving adults (*χ*^2^ = 6.888; *p* < 0.05). Gender played a role in the psychological perception of the responses involving adult patients. The feeling of being overwhelmed during pediatric calls was similar between female respondents (59.9%) and male respondents (58.7%); however, when responding to adult patients, female providers (39.9%) reported distress significantly more frequently than male providers (28.3%) (*χ*^2^ = 8.738; *p* < 0.05). We asked whether the respondents felt confident attending possible future emergencies. The participants’ confidence was significantly lower for pediatric calls (26.9%) compared to adult calls (75.6%) (*χ*^2^ = 354.517; *p* < 0.001). There were no significant differences between EMPs and EMTs or between the genders of the EMS providers regarding confidence with future calls.

The summary of the descriptions of the EMS providers’ most recent calls are summarized in Table 2. The main topics that the EMS providers recalled during calls involving children with LLCs were persistent flashbacks, lack of experience, insufficient communication, and emotional overload. The equivalent issues they recalled for adult patients were discrepancy between the relatives’ wishes and the medical condition of the patient, a desire for contact with palliative care teams, and emotional overload.

**Table 2:** Summary of memories of the participants regarding EMS-calls.

EMS-call for children and adolescents Representative quotes	EMS-call for adults Representative quotes
"It's all a very long time ago. I still find it quite oppressive when I pass by the hospice.”	"Discrepancy between recommendations of emergency doctors and wishes of relatives.”
“Three-year-old child with underlying genetic disease and acute shortness of breath. Can-not-intubate situation, no laryngeal tube available. Catastrophic circumstances.”	“High psychological stress.”
“Chaotic, since little experience of all participants in the field of pediatrics.”	“Often overwhelmed EMS-provider and relatives.”
“Infauste infant resuscitation for known heart defects. Chaotic. >3 years ago.”	“After contact with palliative care team, the situation was much better.”
“As a father, EMS-calls in children are often difficult, because here you also think of your own children and a projection takes place on the sick child.”	“Very stressful.”
“High psychological stress.”	“Depends heavily on the relatives who sometimes, despite palliative care, want the maximum therapy."
“Great uncertainty of the relatives and the EMS.”	
“Often surprising change in primary (phone) diagnosis.”	
“Excessive expectations of the relatives of EMS.”	
“I was pretty overwhelmed."	

**Figure 2:**
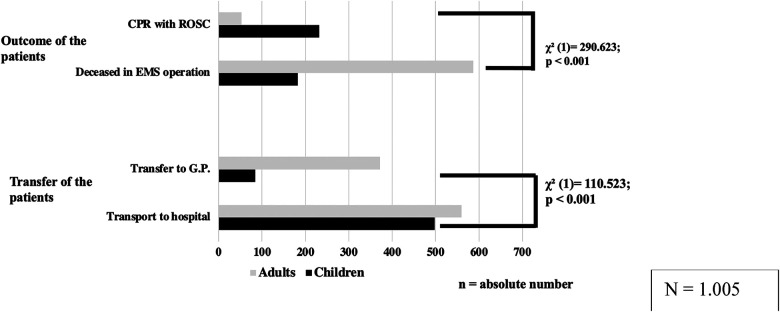
Remembered treatment/outcome of EMS-operations. EMT and EMP reported their experiences regarding the last remembered prehospital EMS- response in children/adults with a life limiting conditions. In outcome adults had a reported significant lower number of ROSC and children were transported more frequently to the hospital. CPR, cardiopulmonary resuscitation; ROSC, recovery of spontaneous circulation; G.P., general practitioner).

Differences were found between the treatment of adults and children in palliative situations (Figure 2). The participants reported transporting children to the hospital significantly more frequently than adults (children: 498/582 [85.6%] respondents; adults: 560/932 [60.1%] respondents; *p* < 0.001). Adult patients were transferred to a GP/family doctor significantly more frequently than children (adults: 372/932 [39.9%] respondents; children: 84/582 [14.4%] respondents; *p* < 0.001).

Regarding the outcome of the patients, adults were reported to have a higher mortality than children (children: 182/582 [31.3%] respondents; adults: 587/932 [63.0%] respondents; *p* < 0.001). Reports of ROSC were significantly more frequent in children than in adults (children: 232/582 [39.9%] respondents; adults: 53/932 [5.7%] respondents; *p* < 0.001).

### Suggestions for treatment in a fictitious case report

**Figure 3:**
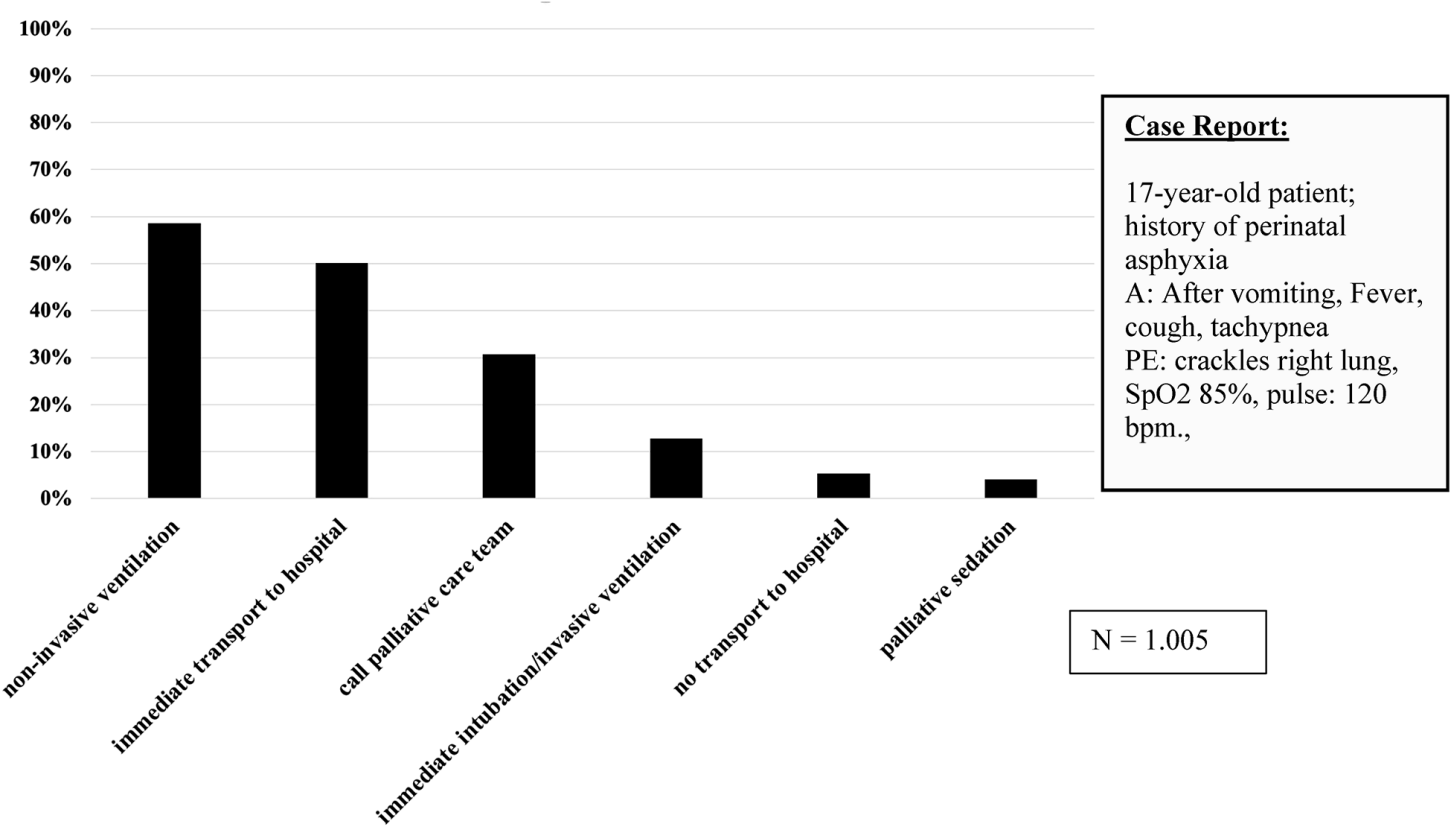
First approaches to case report (multiple selections possible). Responding EMT and EMP tended in the case report to non-invasive ventilation and transport to a hospital. A, anamnesis; PE, physical examination; bpm., beats per minute).

A 17-year-old female patient with a history of severe perinatal asphyxia (GMFCS level V) was presented. The relatives described vomiting beginning 1 day ago. Now, the patient suffers from a fever and cough. The physical examination revealed tachypnea, crackles in the right lung, SpO_2_ 85%, and pulse 120 rpm, indicating pneumonia. The study participants were instructed to provide spontaneous answers regarding treatment suggestions (Figure 3). Of the respondents, 58.6% (*n* = 1,005, MV = 5) stated that they would have preferred non-invasive ventilation whereas 12.8% would have performed intubation and invasive ventilation, 50.1% would have transported the adolescent to a hospital whereas 5.3% would have remained at the patient’s home, and 30.7% would have liked to contact a palliative care team. Overall, 32.8% of EMTs and 33% of male EMS providers chose to contact a palliative care team, in contrast to 23.3% of EMPs and 23.6% of female EMS providers (*χ*^2^ = 7.638/ *χ*^2^ = 8.288; *p* < 0.05).

### Theoretical skills in palliative care

The respondents were asked whether they were familiar with the legal claim for palliative care (which exists in Germany since 2007). In Germany patients with life-limiting conditions suffering from symptoms that cannot sufficiently be controlled in primary or secondary care are entitled to receive pediatric specialized home palliative care covered by statutory health insurance. Only 24.3% and 24.0% gave the correct answer for adults and children, respectively. Of all the respondents, 58.3% reported at least one case in which they contacted a palliative care team for adults, and only 9.7% reported having contacted a palliative care team for children [*χ*^2^ (4) = 83.158; *p* < 0.001; Table 3]. Only 34.5% were familiar with the palliative care team for adults and 9% were familiar with the team for children in their region [*χ*^2^ (4) = 510.049; *p* < 0.001; Table 3].

**Table 3:** Experience and knowledge in palliative care.

Question	Response [*n*/ (%)]	Diff.
Q1: Have you ever had a direct contact to a palliative care team?	A1: “yes” (MV = 12): • team for adults: 586 (58.3%) • team for children: 98 (9.7%)	χ^2^ (4) = 83.158:*p* < 0.001
Q2: Do you know the palliative care team of your region?	A2: “yes” (MV = 33) • team for adults: 347 (34.5%) • team for children: 91 (9.0%)	χ^2^ (4) = 510.049:*p* < 0.001
Q3: Do you think patients have a legal claim for palliative home care?	A3: “yes” (= correct; MV = 467) • care for adults: 244 (24.3%) • care for children: 241 (24.0%)	n.s.
Q4: As an EMS-provider, do you feel yourself responsible in emergencies even for patients (adults and children) under palliative home care?	A4: all answers (MV = 25) • Yes: 744 (75.8%) • No: 88 (9.0%) • Don't know: 149 (15.2%)	
Q5: Do you know tasks of palliative care teams?	A5: (all answers = correct; multiple selections possible; MV = 3) • End-of-life care: 907 (90.1%) • Pain management: 919 (91.4%) • Psychosocial care: 867 (86.4%) • Empower patient's autonomy: 632 (62.8%) • Organization of medical devices: 554 (55.2%) • Advance-care-planning: 535 (53.2%)	
Q6: Which option would you prefer in contacting a palliative care team?	A6: (multiple selections possible; MV = 3) • Standardized folder with medical history/contacts: 840 (83.5%) • Direct contact patched by the 911 coordinator: 562 (55.9%) • Phone number on a patients medical bracelet: 265 (26.3%)	

MV (n), numbers of missing values; Diff., statistically significant difference?

When questioned about the fundamental responsibilities of palliative care teams, 53.2% of the EMS providers provided correct answers regarding advanced care planning and 91.4% provided correct answers regarding pain management. EMPs provided correct answers regarding most of the tasks of the palliative care team: pain management [EMPs 97.7%, EMTs 90%; *χ*^2^ (1) = 13.356; *p* < 0.001], psychosocial care [EMPs 90.8%, EMTs 85.3%; *χ*^2^ (1) = 4.415; *p* < 0.05], empower patient's autonomy [EMPs 86.2%, EMTs 57.1%; *χ*^2^ (1) = 60.192; *p* < 0.001], organization of medical devices [EMPs 67%, EMTs 52.4%; *χ*^2^ (1) = 14.681; *p* < 0.001], and advanced care planning [EMPs 64.7%, EMTs 50.3%; *χ*^2^ (1) = 14.104; *p* < 0.001]. No significant differences were noted between the genders of the EMS providers regarding their responses.

### Demands, needs, and attitudes of the EMS providers

**Figure 4:**
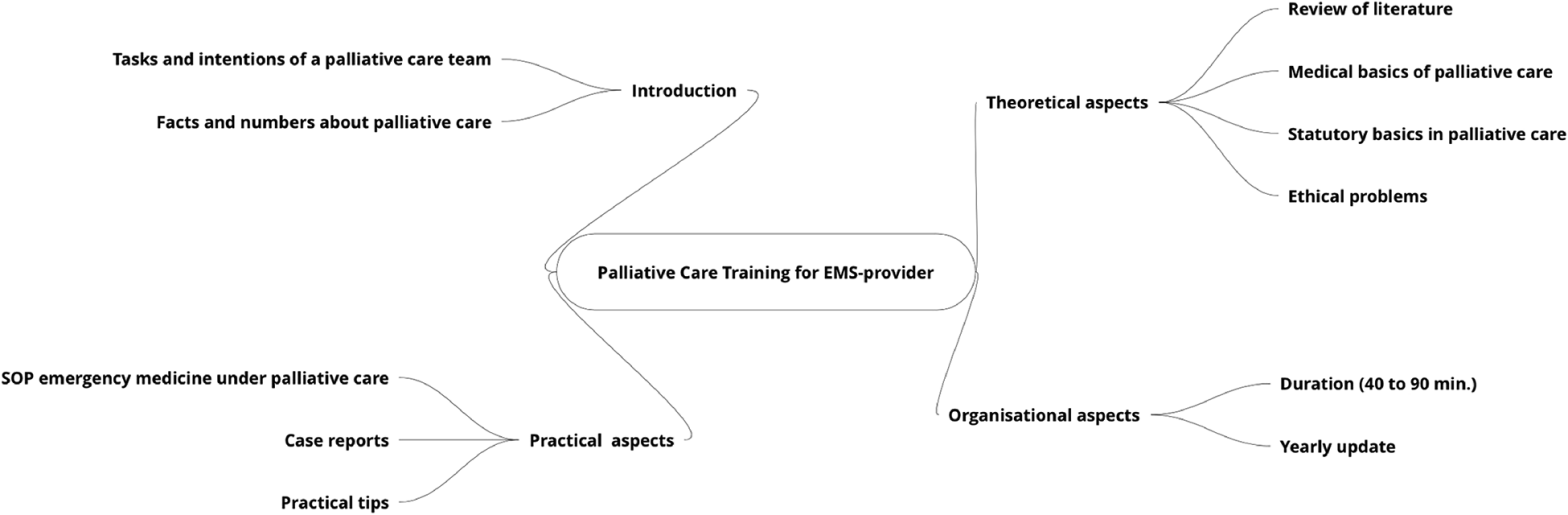
Needs for a special pediatric palliative care training/summary of participant survey.

Overall, 75.8% of the EMS providers felt responsible for all patients with LLCs in emergency situations, with a greater proportion of EMPs feeling responsible than EMTs [EMPs 82.2%, EMTs 73.9%; *χ*^2^ (1) = 9.852; *p* < 0.01]. Most of the EMTs (94.9%) and EMPs (92.4%) desired specific training in palliative care. The duration of this advanced education was recommended to be a mean 90 ± 72 min by EMPs and 166 ± 119 min by EMTs (*p* < 0.001). No differences were observed in the desired topics of the palliative care training between EMS providers (Figure 4). In summary, the introduction of the training should be short and should lead to practical issues, such as case reports and standard operating procedures. The respondents’ theoretical needs were interest in basic literature (more in EMPs) and ethical problems regarding the wishes of relatives compared with the possible medical outcomes. A yearly update of the training was requested by most of the EMS providers.

## Discussion

This study provides insight into the interface between the EMS and palliative home care, with a focus on children and adolescents. The traditional key intention of the EMS is to save lives through resuscitation, while palliative care providing help in reducing the distressful symptoms of LLC. These purposes are not contradictory, and a pediatric emergency involving a child with LLCs is a significant challenge for both groups of caregivers. The literature contains several studies and two recently published reviews on EMS and palliative care focusing on the perceptions of the role of EMS providers in palliative care, understanding the patients’ wishes, and supporting patients’ families (24). Other topics include the process of decision making, providing complex care, insufficient support of palliative care, and grief (21, 25). Data, especially on children with LLCs, are lacking. One pediatric interview study with 22 participants (13) focused on the interface between families and the EMS and revealed varying confidence levels and a preference for advance health directives from the primary physician provided by the family for guiding the treatment in emergencies.

### Open interviews with EMS supervisors

The interviews were helpful in revealing new topics for the questionnaire. Due to the lack of data on emergencies involving children with LLCs regarding the emotional aspects for EMS providing for a dying child, it was important to ask the EMS providers’ experiences and strategies of relief from the resulting consequences. Another key finding was the lack of training in palliative care. It was observed across all three levels of German EMT education. The interviews in this study revealed similar findings to those of Breyre regarding emotional overload (25, 26); however, our study revealed a significantly greater emotional burden in emergencies involving children with life-limiting conditions.

### Demographics

A strength of the questionnaires was a comprehensive sample size of >1,000 participants (27–30), with a high response rate of >80%, representing all levels of EMT and EMP education and providing broad insight into the experiences of EMS providers. The higher age of EMPs may be due to the longer duration of medical studies (6 years) compared with EMT training (520 h to 3 years).

### Experiences of EMS providers

In recent years, publications have indicated a growing awareness of EMS providers regarding patients with life limiting conditions (31–33). In this study >90% of providers could recall an EMS call involving adults with LLCs, which was expected. However, surprisingly, also 60% of the respondents reported that they had experienced at least one emergency call involving children with a LLC. Recently, a retrospective study from Queensland, Australia reported 4,348 cases of adults with LLCs (0.5% of), of which 3,237 cases (74.4% of all patients with life-limiting conditions) were transported to a hospital within the 1-year study period (33). Furthermore, 90.1% of the respondents in a study by Wiese et al. reported a similar rate of a contact with adults with LLCs (34). Therefore, EMS providers may have more experience with adults and less experience with children and adolescents. In a retrospective study, 3.9% of the analyzed 1,583 EMS calls involving palliatively treated patients revealed that only 0.2% calls involved children under palliative care (14). One factor that might have contributed to the higher rate of pediatric calls in our study could be the high mean job experience of more than 11 years (range: 0–42 years) of the participants in the study. Furthermore, bias due to social desirability or varying awareness of the definition of “palliative” in children cannot be excluded.

Treatment of children in emergencies is psychologically demanding, especially in a palliative setting. Pediatric calls are reported to be significantly more overwhelming than adult emergencies (9). This is in line with interview study from Eich et al. (12) where 43 emergency physicians reported experiencing the greatest deficits in the care of infrequent but life-threatening emergencies involving children. The reasons for the same deficits identified in our study were described as missing routine, emotional aspects, and psychological overload (Table 2). Furthermore, the study participants of our study reported being overwhelmed with the EMS call in 31% in an adult and 60% in a pediatric palliative setting. Regarding future pediatric calls, the providers’ confidence was significantly lower compared to adult calls. To our knowledge, there are no published data comparing EMS calls involving palliatively treated children with those involving adults under palliative care. In a multicenter Turkish study, 570 participants explained their burnout symptoms as being caused by emotional exhaustion in >60% (35). Mishra et al. reported that, in EMS personnel (*N* = 105), 83% reported experiencing symptoms and 5% met the clinical criteria of post-traumatic stress disorder (PTSD) (36). This is evidence that caring for critically ill children results in significant psychological burden on the caretakers, and our findings confirm that emergency medicine professionals are equally affected. The free answers of the EMS providers expressed their challenges and were in accordance with the results of the closed questions and rating scales.

We asked the participants about the therapy they performed in children and adults with LLCs. In our data, the reported number of transports to the hospital was higher in children than in adults. There was a significantly higher reported number of ROSCs in children compared to adults, while a higher number of adults were reported as deceased. This could be due to a higher incidence of comorbidities (e.g., cardiovascular diseases, cancer, age >80 years) in adults in general, which is consistent with the reports of adult CPR studies (37–39). In our data, there was a lower rate of GPs being involved in pediatric cases. This may be due to the broader coverage of medical care in adults. In Germany, children are typically treated by pediatricians. In contrast with GPs and EMS providers, pediatricians are not present 24/7 and do not routinely perform home visits (40).

### Suggestions for treatment in a fictitious case report

This case was chosen because this is a typical case of an EMS response in children receiving palliative home care (4). It was also chosen, because there was no right or wrong response. However, this case has the potential to provide insights into the attitudes of the EMS providers. The answers involving life-sustaining actions were threefold higher than pure palliative responses and may reflect the common practice. It will be interesting to evaluate whether an education program for EMS personnel will influence the providers’ management of future cases. Furthermore, the variety of different approaches indicates that there is a need for new guidelines for the EMS regarding decision-making processes in emergencies.

### Theoretical skills in palliative care

In our study, we also discovered a lack of theoretical knowledge. The legal claim for palliative home care was unknown by most of the participants; moreover, <10% were informed about the availability of palliative home care for children.

### Demands, needs, and attitudes of EMS providers

A mixed methods study by Shearer from Perth, Australia, including 66 participants concluded that EMS personnel sought further education in communication and ethics to reduce their lack of confidence (41). In our study also more than 90% of participants desired special EMS training for palliative situations. Another important finding was that the study participants felt responsible for the patients in emergency situations even though they were treated by a 24/7 palliative home care team. This is consistent with a qualitative study by Hoare from Cambridge, UK, where interviewees were broadly consistent in enabling adults to die at home (42). In a Canadian study, Jensen revealed that a program for extended-care paramedics who care for long-term care residents can play a key role in end-of-life care (43).

In summary, we found a lack of specific programs focusing on the special needs of children receiving palliative home care. As a consequence, a specific pediatric education program will be implemented in the reporting region of Hesse, Germany. An important part of the future guideline is to inform the participants about the importance and the limitations of decision making. DNR orders or Physician Order for Life-Sustaining Treatment (POLST) forms can help in making these decisions (26). In a qualitative study on a group of 15 EMTs and paramedics, the participants stated that formal training was needed to better prepare them for the emotional aspects of communicating with patients and their families, to handle grief, and to obtain advice in decision making (25). However, neither study focused on children or adolescents.

In time-critical situations, it is important that the EMS is rapidly available as these providers are still needed despite 24/7 on-call palliative care. A study by Wiese et al. revealed that the involvement of an SHPC team in adult cases led to a lower rate of emergency calls to the EMS (44). Cooperation at the sensitive interface between emergency services and SHPC teams is urgently needed for this highly vulnerable group of children and adolescents.

### Limitations and strengths of the study

This study is limited to the experiences of German EMS teams with a system involving prehospital EMPs. The answers of the participants regarding the reported number of EMS calls involving children with LLCs cannot be validated and might have been influenced by the bias of social desirability. Due to the design of this study, it was not possible to estimate the prevalence of children with LLCs in the EMS system. To our knowledge, this is the first study exploring the role of the EMS with a large number of participants.

## Conclusions

Children with LLCs appear to play an underestimated role in EMS. The treatment of children with severe, chronic, and rare diseases is challenging, and specialized training should be offered to EMS professionals. We believe that a training program is the first step to facilitating the necessary cooperation in the interface between palliative care and EMS.

## Data Availability

The raw data supporting the conclusions of this article will be made available by the authors, without undue reservation.
